# Multi-Substrate Terpene Synthases: Their Occurrence and Physiological Significance

**DOI:** 10.3389/fpls.2016.01019

**Published:** 2016-07-12

**Authors:** Leila Pazouki, Ülo Niinemets

**Affiliations:** ^1^Department of Plant Physiology, Institute of Agricultural and Environmental Sciences, Estonian University of Life SciencesTartu, Estonia; ^2^Estonian Academy of SciencesTallinn, Estonia

**Keywords:** monoterpene synthesis, multi-substrate terpene synthases, prenyltransferases, sesquiterpene synthesis, subcellular compartmentalization, terpene engineering, terpene metabolites

## Abstract

Terpene synthases are responsible for synthesis of a large number of terpenes in plants using substrates provided by two distinct metabolic pathways, the mevalonate-dependent pathway that is located in cytosol and has been suggested to be responsible for synthesis of sesquiterpenes (C15), and 2-C-methyl-D-erythritol-4-phosphate pathway located in plastids and suggested to be responsible for the synthesis of hemi- (C5), mono- (C10), and diterpenes (C20). Recent advances in characterization of genes and enzymes responsible for substrate and end product biosynthesis as well as efforts in metabolic engineering have demonstrated existence of a number of multi-substrate terpene synthases. This review summarizes the progress in the characterization of such multi-substrate terpene synthases and suggests that the presence of multi-substrate use might have been significantly underestimated. Multi-substrate use could lead to important changes in terpene product profiles upon substrate profile changes under perturbation of metabolism in stressed plants as well as under certain developmental stages. We therefore argue that multi-substrate use can be significant under physiological conditions and can result in complicate modifications in terpene profiles.

## Introduction

Plants synthesize a vast array of secondary metabolites, many of which have been used by humans due to their medicinal, culinary, and cosmetic properties (Balandrin et al., 1985). Terpenoids with different molecular size and very high structural diversity constitute the largest group of plant secondary metabolites with more than 60,000 (http://dnp.chemnetbase.com) representatives (Cheng et al., 2007; Xie et al., 2012). Terpenes are synthesized in all known organismal groups where they fulfill a plethora of functions ranging from primary metabolism to antioxidative and structural functions (Pontin et al., 2015). They are extensively explored for their diverse applications as agricultural chemicals, flavors and fragrances, medicines and industrial chemicals (Pontin et al., 2015).

All terpenes are derived from C5 building blocks, isopentenyl diphosphate (IDP) and dimethylallyl diphosphate (DMADP). They are produced by two distinct pathways, the mevalonate (MVA) pathway, which functions in archaea and in some bacteria and in the cytosol of plants, animals and fungi, and the 2-C-methyl-D-erythritol-4-phosphate (MEP) pathway present in most bacteria and in plant plastids and affiliated organelles such as apicoplasts in Apicomplexa (Smit and Mushegian, 2000; Degenhardt et al., 2009; Nagegowda, 2010; Lombard and Moreira, 2011). IDP and DMADP are further condensed by enzymes called prenyltransferases resulting in a multitude of intermediates with different chain length including geranyl diphosphate (GDP, C10), farnesyl diphosphate (FDP, C15), geranylgeranyl diphosphate (GGDP, C20), and squalene (C30; Koyama and Ogura, 1999; Lange et al., 2000; Rodríguez-Concepción, 2006). These intermediates are further used by a large class of enzymes called terpene synthases (TPSs) including hemiterpene synthases responsible for formation of hemiterpenes isoprene and 2-methyl-butenol (C5), monoterpene synthases for monoterpenes (C10), sesquiterpene synthases for sesquiterpenes (C15), and diterpene synthases for diterpenes (C20; Bohlmann and Croteau, 1999; Chen et al., 2011). A peculiar feature of TPSs is that they first form a highly reactive substrate carbocation that is further rapidly converted to different carbocation intermediates, typically giving rise of multiple terpene products (Bohlmann and Keeling, 2008; Christianson, 2008). The **product specificity** of different terpenoid synthases is very variable and primarily depends on how well the substrate carbocation can be stabilized in the enzyme active center (Bohlmann and Keeling, 2008; Christianson, 2008).

KEY CONCEPT 1 **Product specificity.**

Capacity to form specific reaction products. Terpene synthases typically form multiple products, but the diversity of products varies for different terpene synthases with some catalyzing synthesis of a limited number of products (high product specificity) and others catalyzing a large variety of different terpenes (low product specificity). Multi-substrate enzymes always form different products with different substrates, but this concept refers to the diversity of products formed with given substrate.

Recent progress in cloning of multiple terpene biosynthesis genes, expression in heterologous systems, and functional characterization of corresponding enzymes have greatly contributed to improve understanding of functions of TPSs and regulation of genes involved in terpene synthesis pathways both in angiosperms and gymnosperms (Martin et al., 2004; Keeling and Bohlmann, 2006; Degenhardt et al., 2009; Chen et al., 2011; Pazouki et al., 2015). In plants harboring two largely independent pathways for production of terpene precursors, MVA and MEP pathways, recent work has opened up an exciting novel and so far hidden aspect of regulation of terpene synthesis that challenges the current consensus on the compartmentalization and regulation of terpene synthesis. In particular, there is evidence that several TPSs are **multi-substrate** enzymes, capable of synthesizing terpenes of different chain length depending on corresponding substrate availability (Davidovich-Rikanati et al., 2008; Gutensohn et al., 2013; Pazouki et al., 2015). Among such multi-substrate enzymes, some can form monoterpenes with GDP as the substrate and sesquiterpenes with FDP as the substrate (Davidovich-Rikanati et al., 2008; Gutensohn et al., 2013; Pazouki et al., 2015).

KEY CONCEPT 2 **Multi-substrate terpene synthases.**

TPSs that can use prenyl diphosphates with different chain length or different *cis*/*trans* configuration as substrates.

**Figure 1:**
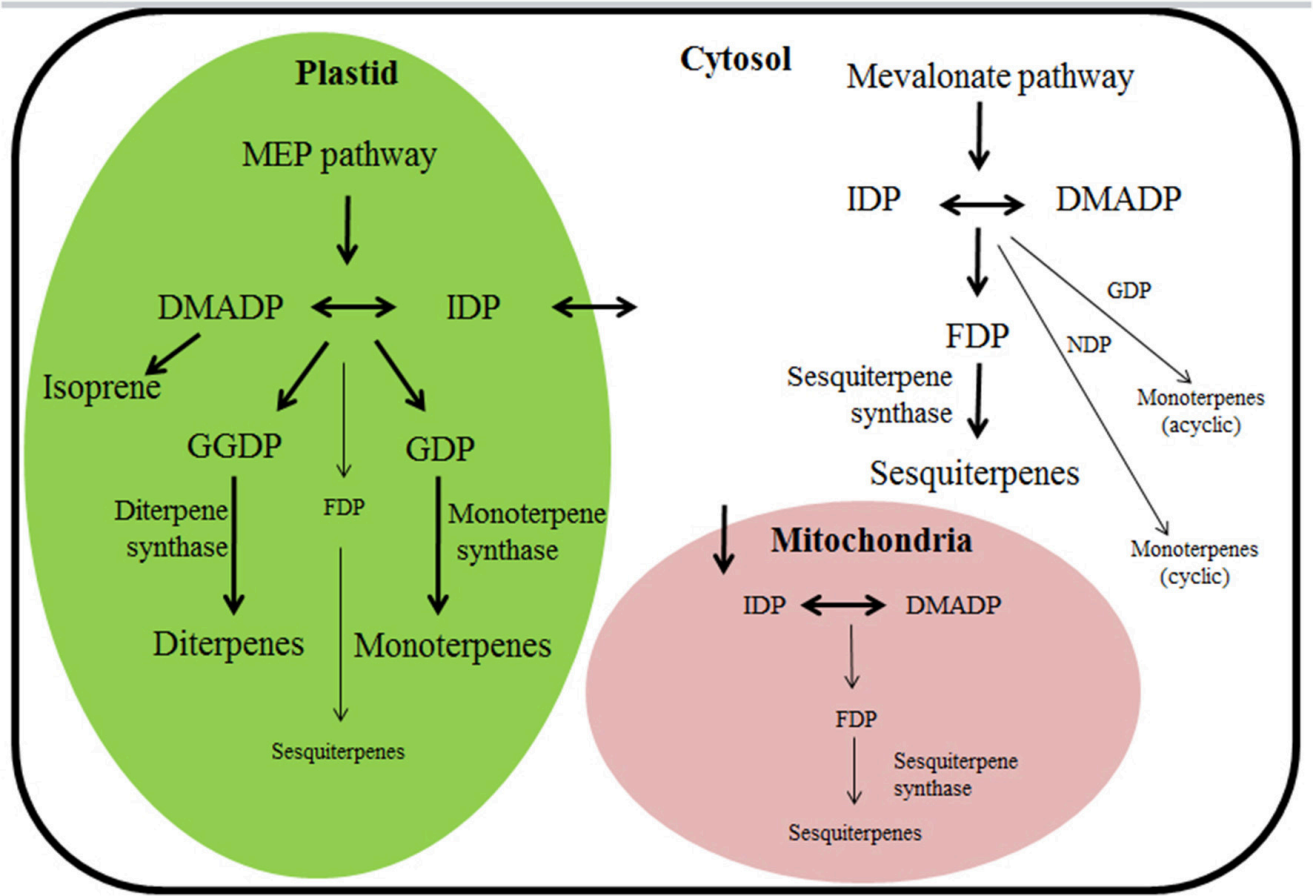
**Terpene biosynthetic pathways and their subcellular compartmentalization in plants**. Thick arrows denote the classical understanding of terpenoid synthesis compartmentalization among cytosol and plastid (Bohlmann et al., 1998b; Chen et al., 2011; Tholl and Lee, 2011), reflecting the circumstance that monoterpene and diterpene synthases harboring a chloroplast-targeting peptide are functionally active in plastids and sesquiterpene synthases lacking the target peptide are active in cytosol. However, recent findings of the capacity for multi-substrate use of several mono, sesqui-, and, diterpene synthases suggest that when substrate becomes available, several cytosolic “sesquiterpene” synthases could also operate as monoterpene synthases, and analogously, multi-substrate “monoterpene” and “diterpene” synthases could operate as sesquiterpene synthases in plastids (denoted by thin arrows). In addition, terpenoid synthesis can also potentially occur in mitochondria (Nagegowda, 2010; Tholl and Lee, 2011; Dong et al., 2016). For instance, targeting linalool/(*E*)-nerolidol synthase (FaNES1) from *Fragaria ananassa* (Table 1 for protein specifics) to the mitochondria led to the production of (*E*)-nerolidol and homoterpene 4,8-dimethyl nona-1,3,7-triene (DMNT) in transgenic *Arabidopsis thaliana* plants (Kappers et al., 2005). DMADP, dimethylallyl diphosphate (C5); MEP pathway, 2-C-methyl-D-erythritol 4-phosphate/1-deoxy-D-xylulose 5-phosphate pathway; IDP, isopentenyl diphosphate (C5); FDP, farnesyl diphosphate (C15); GDP, geranyl diphosphate (C10); GGDP, geranylgeranyl diphosphate (C20); NDP, neryl diphosphate (C10).

The biological significance of the finding of multi-substrate use has been debated as according to the current consensus, hemiterpene, monoterpene, and diterpene syntheses are confined to plastids and rely on substrates provided by the MEP pathway, while sesquiterpene synthesis is confined to cytosol and relies on substrates provided by the MVA pathway (Figure 1; Dudareva et al., 2004, 2006; Keeling et al., 2008). However, there has been significant progress in understanding the subcellular distribution of substrates with differing chain length and **cross-talk between the two pathways** for substrate formation (Gutensohn et al., 2013; Rasulov et al., 2015; Dong et al., 2016). Since in plants both pathways (MEP and MVA) synthesize the same substrates, DMADP and IDP, there has been a long-standing enigma as to whether the two pathways can exchange metabolites (Rodriguez-Concepcion and Boronat, 2002). A certain exchange of IDP between cytosolic and plastidic compartments has been considered as the most likely point of convergence of the two pathways (Schwender et al., 2001; Bick and Lange, 2003). Although the overall intercompartmental exchange of terpene substrates from one compartment to pathway flux in the other subcellular compartment is minor under non-stressed conditions, the importance of cross-talk among the pathways might increase under stress conditions that particularly suppress terpene synthesis in one pathway or under certain developmental stages (Dudareva et al., 2005; Maya et al., 2013; Rasulov et al., 2015). Furthermore, substrate exchange at the level of larger isoprenoids such as GDP has been also shown to be possible (Bick and Lange, 2003; Dong et al., 2016). In fact, several recent reports demonstrate that monoterpenes can be synthesized by multi-substrate sesquiterpene synthases in the cytosol (Davidovich-Rikanati et al., 2008; Gutensohn et al., 2013). Such a multi-substrate use capacity can provide an alternative means for regulation of mono- and sesquiterpene production through modification of cytosolic pool sizes of different substrates. On the other hand there is evidence of sesquiterpene production in plastids (Van Schie et al., 2007; Nagegowda, 2010). Furthermore, mitochondria could potentially contribute to both mono- and sesquiterpene synthesis (Figure 1, Tholl and Lee, 2011; Dong et al., 2016).

KEY CONCEPT 3 **Cross-talk among plastidic and cytosolic isoprenoid synthesis pathways.**

Plants have two isoprenoid synthesis pathways, 2-C-methyl-D-erythritol 4-phosphate pathway (MEP pathway) and mevalonate (MVA) pathway that were assumed to operate independently. There is now evidence that the pathway products, in particular, C5 intermediate isopentenyl diphosphate (IDP), and C10 intermediate geranyl diphosphate (GDP) and possibly also C15 intermediate farnesyl diphosphate (FDP) can be exchanged between plastids and cytosol, indicating that the two pathways are not totally independent.

A possible multi-substrate use in both plastids and cytosol opens up a previously non-considered opportunity of modification of terpene product profiles by changes in pool sizes of substrates with different chain length. This could be of particular importance for aromatic plants that lack specialized terpene storage structures. Because no time-consuming gene expression is needed, only changes in substrate pool sizes could result in rapid alteration of the small bouquets in such species. In fact, a capacity of multi-substrate use can be more widespread than currently recognized, because so far, functional characterization of TPSs is often conducted with only a single substrate or limited range of substrates (Rajabi et al., 2013).

This focused review highlights the widespread presence of multi-substrate terpenes, analyzes their evolutionary relationships, and physiological significance with special emphasis on subcellular localization of multi-substrate TPSs and the possible availability of corresponding substrates. We argue that this understudied facet of terpenoid metabolism plays a significant role in determining terpene profiles in natural conditions.

## Widespread multi-substrate use of TPSs in plants

The first multi-substrate enzyme described was a (*E*,*E*)-β-farnesene synthase from the aromatic herb *Mentha x piperita* (Crock et al., 1997). It is one of the simplest sesquiterpene synthases that uses FDP to produce mainly (*E*,*E*)-β-farnesene (85%) and lower amounts of (*Z*)-β-farnesene (8%) and δ-cadinene (5%). It can also use GDP as substrate and produce several different cyclic monoterpene products such as limonene (48%) and terpinolene (15%) and the acyclic monoterpene myrcene (15%), but this enzyme lacks a N-terminal transit peptide (Crock et al., 1997). Shortly afterwards, α-bisabolene synthase from the gymnosperm tree *Abies grandis* was isolated that catalyzes synthesis of sesquiterpene *E*-α-bisabolene with FDP and monoterpene (+)-limonene with GDP (Bohlmann et al., 1998b). This enzyme also lacked the transit peptide and had a greater sequence similarity to other *A. grandis* sesquiterpene synthases, δ-selinene synthase and γ-humulene synthase, than to *A. grandis* monoterpene synthases, suggesting that (*E*)-α-bisabolene synthase gene encodes a sesquiterpene synthase (Bohlmann et al., 1998b). In contrast, *A. grandis* δ-selinene synthase and γ-humulene synthase are similar to *E*-α-bisabolene synthase in their capacity to produce monoterpenes when incubated with GDP (Table 1; Steele et al., 1998). Analogously, germacrene C synthase from *Solanum lycopersicum* clone pLE11.3 was also shown to accept more than one substrate, producing germacrene C (64%), germacrene A (18%), germacrene B (11%), and germacrene D (7%) with FDP, and limonene with GDP (Colby et al., 1998).

**Table 1:** **Overview of plant terpene synthases with confirmed multi-substrate activity**.

**Terpene synthase**	**GenBank accession number**	**Species**	**TPS family**	**Presence of transit peptide^a^**	**Substrate^b^**	**Terpenoid products^c^**	**References**
Germacrene A synthase (AmGAS)	KC145534	*Achillea millefolium*	TPS-a	N	(*E,E*)-FDP	Germacrene A, β-elemene, β-selinene, α-selinene	Pazouki et al., 2015
					GDP	Myrcene, (*E*)-β-ocimene, (*Z*)-β-ocimene, limonene, terpinolene, α-pinene, camphene	
					NDP	2-Carene, γ-terpinene, α-terpinene, α-fenchene, α-thujene	
Myrcene synthase	EU760349	*Humulus lupulus*	TPS-b	Y	DMADP	Isoprene	Sharkey et al., 2013
					GDP	Myrcene	
Linalool synthase (LeMTS1)	JN408286	*Solanum lycopersicum*	TPS-b	Y	GDP	Linalool	Van Schie et al., 2007
					(*E,E*)-FDP	(*E*)-Nerolidol	
Nerolidol synthase (FaNES1)	AX528996	*Fragaria ananassa*	TPS-g	N	(*E,E*)-FDP	(*E*)-Nerolidol	
					GDP	Linalool	
Nerolidol synthase (FaNES2)	AX529067	*Fragaria ananassa*	TPS-g	Y	(*E,E*)-FDP	(*E*)-Nerolidol	Aharoni et al., 2004
					GDP	Linalool	
Nerolidol synthase (FvNES1)	AX529002	*Fragaria vesca*	TPS-g	Y	(*E,E*)-FDP	(*E*)-Nerolidol	Aharoni et al., 2004
					GDP	Linalool	
α-farnesene synthase	AY787633	*Malus × domestica*	TPS-b	N	(*E,E*)-FDP	α-farnesene, β-farnesene	Green et al., 2007
					GDP	Linalool, (*Z*)-β-ocimene, (*E*)-β-ocimene, β-myrcene	
					GDP and IDP	α-farnesene	
Santalene synthase (SaSSy)	HQ343276	*Santalum album*	TPS-b	N	(*E,E*)-FDP	α-Santalene, β-santalene, *epi*-β-santalene, α-*exo*-bergamotene, α-farnesene (traces), β-farnesene (traces)	Jones et al., 2011
					(*Z,Z*)-FDP	α-*endo*-Bergamotene, α-santalene, (*Z*)-β-farnesene, *epi*-β-santalene, β-santalene	
					GDP	Linalool, geraniol, terpineol, α-pinene (traces), camphene (traces)	
Santalene synthase (SspiSSy)	HQ343278	*Santalum spicatum*	TPS-b	N	(*E,E*)-FDP	α-Santalene, β-santalene, *epi*-β-santalene, α-*exo*-bergamotene, α- farnesene (traces), β- farnesene (traces)	Jones et al., 2011
					GDP	Linalool, geraniol, terpineol, α-pinene (traces), camphene (traces)	
Santalene synthase (SauSSy)	HQ343277	*Santalum austrocaledonicum*	TPS-b	N	(*E,E*)-FDP	α-Santalene, β-santalene *epi*-β-santalene, α-*exo*-bergamotene, α-farnesene (traces), β-farnesene (traces)	Jones et al., 2011
					GDP	Linalool, geraniol, terpineol, α-pinene (traces), camphene (traces)	
β-Bisabolene synthase	HQ343279	*Santalum austrocaledonicum*	TPS-a	N	(*E,E*)-FDP	β-Bisabolene, α-bisabolol (traces)	Jones et al., 2011
					GDP	Limonene, terpineol	
Sesquiterpene synthase (SspiSesquiTPS)	HQ343282	*Santalum spicatum*	TPS-a	N	(*E,E*)-FDP	β-elemol, guaiol, bulnesol	Jones et al., 2011
					GDP	Linalool (traces)	
Sesquiterpene synthase (SauSesquiTPS)	HQ343281	*Santalum austrocaledonicum*	TPS-a	N	(*E,E*)-FDP	α-Humulene, δ-cadinene, β-elemene	Jones et al., 2011
					GDP	Linalool (traces)	
Monoterpene synthase (SaMonoTPS1)	JF746815	*Santalum album*	TPS-b	N	(*E,E*)-FDP	β-Bisabolene, α-bisabolol (traces)	Jones et al., 2008
					GDP	Limonene, α -terpineol	
(*E*)-β-farnesene synthase	AF024615	*Mentha x piperita*	TPS-a	N	(*E,E*)-FDP	(*E*)-β-farnesene, (*Z*)-β-farnesene, δ-cadinene	Crock et al., 1997
					GDP	Limonene, terpinolene, myrcene	
(*E,E*)-α-farnesene synthase	AY640154	*Cucumis sativus*	TPS-a	Y	(*E,E*)-FDP	(*E,E*)-α-farnesene	Mercke et al., 2004
					GDP	(*E*)-β-ocimene	
α-Zingiberene synthase (ZIS)	AY693646	*Ocimum basilicum*	TPS-b	N	(*E,E*)-FDP	α-Zingiberene, (*Z*)-α-bergamotene, (*E*)-α-bergamotene, 7-*epi*-sesquithujene, sesquithujene, β-sesquiphellandrene, (*Z*)-β-farnesene, (*E*)-β-farnesene, β-bisabolene, α-acoradiene, β-curcumene	Davidovich-Rikanati et al., 2008; Gutensohn et al., 2013
					GDP	α-Thujene, α-pinene, β-phellandrene, γ-terpinene, *p*-cymene	
Nerolidol/linalool synthase (AmNES/LIS1)	EF433761	*Antirrhinum majus*	TPS-g	N	(*E,E*)-FDP	(*E*)-Nerolidol, β-farnesene, α-farnesene, α-bisabolol	Nagegowda et al., 2008
					GDP	Linalool, (*E*)-β-ocimene, myrcene, α-pinene	
Nerolidol/linalool synthase (AmNES/LIS2)	EF433762	*Antirrhinum majus*		Y	(*E,E*)-FDP	(*E*)-Nerolidol, β-farnesene, α-farnesene, α-bisabolol	Nagegowda et al., 2008
					GDP	Linalool, (*E*)-β-ocimene, myrcene, α-pinene	
(*E*)-α-Bergamotene synthase (LaBERS)	DQ263742	*Lavandula angustifolia*	TPS-b	N	(*E,E*)-FDP	(*E*)-α-Bergamotene, (*E*)-nerolidol, (*Z*)-α-bisabolene, (*E*)-β-farnesene, β-sesquiphellandrene	Landmann et al., 2007
					GDP	α-Pinene, sabinene, limonene, β-pinene, camphene, β-myrcene	
α-Bisabolene synthase	AF006194	*Abies grandis*	TPS-d	N	(*E,E*)-FDP	(*E*)-α-Bisabolene	Bohlmann et al., 1998b
					GDP	(+)-Limonene	
Germacrene C synthase (clone pLE11.3)	AF035630	*Solanum lycopersicum* cv. VFNT	TPS-a	N	(*E,E*)-FDP	Germacrene C, germacrene A, germacrene B, germacrene D	Colby et al., 1998
					GDP	Limonene	
δ-Selinene synthase	AGU92266	*Abies grandis*	TPS-d	N	(*E,E*)-FDP	34 different sesquiterpenes with δ-selinene, germacrene B, guaia-6,9-diene, germacrene A and δ-amorphene as the main products	Steele et al., 1998
					GDP	Limonene, (*Z*)-b-ocimene, myrcene, terpinolene, (*E*)-b-ocimene, α-terpinene, γ-terpinene, α-pinene, β-pinene, sabinene	
γ-Humulene synthase	AGU92267	*Abies grandis*	TPS-d	N	(*E,E*)-FDP	52 different sesquiterpenes with γ-humulene, sibirene, longifolene, b-himachalene, γ-himachalene and α-himachalene as the main products	Steele et al., 1998
					GDP	Limonene, terpinolene, myrcene, (*E*)-β-ocimene, camphene, α-pinene, β-pinene, sabinene, α-thujene, α-terpinene	
Kaurene synthase like (TaKSL5)	AB597958	*Triticum aestivum*	TPS-e	Y	*ent*-CDP	*ent*-Kaurene	Hillwig et al., 2011
					(*E,E*)-FDP	(*E*)-Nerolidol	
Terpene synthase (PlTPS2)	KC012520	*Phaseolus lunatus*	TPS-g	Y	(*E,E*)-FDP	(*E*)-Nerolidol	Brillada et al., 2013
					GDP	Linalool	
					GGDP	(*E*,*E*)-Geranyllinalool	
Terpene synthase (MtTPS3)	AY766249	*Medicago truncatula*	TPS-g	Y	(*E,E*)-FDP	(*E*)-Nerolidol	Arimura et al., 2008
					GDP	Linalool	
					GGDP	(*E*,*E*)-Geranyllinalool	
Sesquiterpene synthase (Os08g07100)	EU596452	*Oryza sativa* cv. Nipponbare	TPS-b	N	(*E,E*)-FDP	14 sesquiterpenes with zingiberene, β-sesquiphellandrene and (*E*)-γ-bisabolene as main products	Yuan et al., 2008
					GDP	several monoterpenes with β-myrcene as main product	
Terpene synthase (At3g25810)	BT053763	*Arabidopsis thaliana*	TPS-b	Y	(*E,E*)-FDP	(*E,E*)-α-farnesene, (*E*)-β-farnesene, (*E*)-α-bergamotene	Chen et al., 2003
					GDP	α-Pinene, sabinene, β-pinene, β-myrcene, limonene, (*E*)-β-ocimene	
β-ocimene synthase (AtTPS02)	At4g16730	*Arabidopsis thaliana*	TPS-b	Y	(*E,E*)-FDP	(*E,E*)-α-farnesene	Huang et al., 2010
					GDP	(*E*)-β-ocimene	
(*E,E*)-α-farnesene synthase (AtTPS03)	At4g16740	*Arabidopsis thaliana*	TPS-b	N	(*E,E*)-FDP	(*E,E*)-α-farnesene	Huang et al., 2010
					GDP	(*E*)-β-ocimene	
Linalool/Nerolidol synthase (VvPNLinNer1)	HM807391	*Vitis vinifera*	TPS-g	N	(*E,E*)-FDP	(*E*)-Nerolidol	Martin et al., 2010
					GDP	Linalool	
Linalool/Nerolidol synthase (VvPNLinNer2)	HM807392	*Vitis vinifera*	TPS-g	N	(*E,E*)-FDP	(*E*)-Nerolidol	Martin et al., 2010
					GDP	Linalool	
Linalool/Nerolidol synthase (VvCSLinNer)	HM807393	*Vitis vinifera*	TPS-g	N	(*E,E*)-FDP	(*E*)-Nerolidol	Martin et al., 2010
					GDP	Linalool	
Linalool/(*E*)- nerolidol/(*E*,*E*)-geranyllinalool synthases (VvPNLNGl1-VvPNLNGl4)	HM807394	*Vitis vinifera*	TPS-g	N	(*E,E*)-FDP	(*E*)-Nerolidol	Martin et al., 2010
(*E*)-Nerolidol/(*E,E*)-geranyllinalool synthase (VvCSENerGl)	HM807400	*Vitis vinifera*	TPS-f	N	(*E,E*)-FDP	(*E*)-Nerolidol	Martin et al., 2010
					GGDP	(*E*,*E*)-Geranyllinalool	
(*E*)-Nerolidol/(*E,E*)-geranyllinalool synthase (VvPNENerGl)	HM807401	*Vitis vinifera*	TPS-f	N	(*E,E*)-FDP	(*E*)-Nerolidol	Martin et al., 2010
					GGDP	(*E*,*E*)-Geranyllinalool	
(*E*)-β-ocimene/(*E,E*)-α-farnesene synthase (VvGwbOciF)	HM807388	*Vitis vinifera*	TPS-b	N	(*E,E*)-FDP	(*E,E*)-α-farnesene	Martin et al., 2010
					GDP	Linalool	
(*E*)-β-ocimene/(*E,E*)-α-farnesene synthase (VvCSbOciF)	HM807389	*Vitis vinifera*	TPS-b	N	(*E,E*)-FDP	(*E,E*)-α-farnesene	Martin et al., 2010
					GDP	Linalool	
Linalool/Nerolidol synthase (VvRILinNer)	JQ062931	*Vitis vinifera*	TPS-g	Y	(*E,E*)-FDP	(*E*)-Nerolidol	Zhu et al., 2014
					GDP	Linalool	

a *Y, transit peptide is present; N, transit peptide is absent*.

b *DMADP, dimethylallyl diphosphate (C5); IDP, isopentenyl diphosphate (C5); GDP, geranyl diphosphate (C10); FDP, farnesyl diphosphate (C15); NDP, neryl diphosphate (C15); CDP, copalyl diphosphate (C20); GGDP, geranylgeranyl diphosphate (C20)*.

c *products synthesized from given substrates are ordered according to the relative importance in the product blend*.

**Figure 2:**
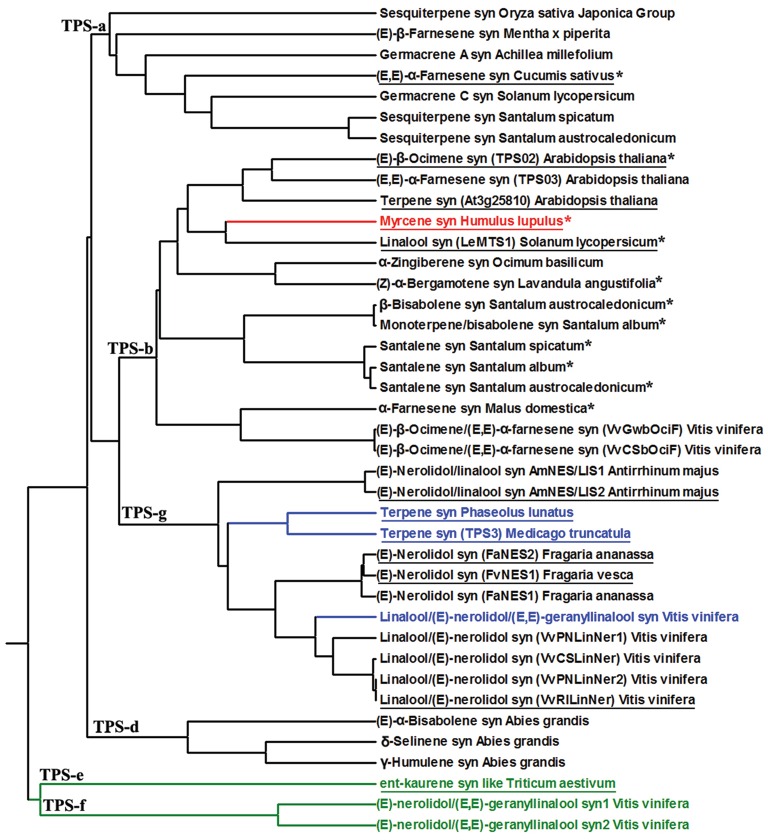
**Phylogenetic tree of terpene synthases (TPS) with confirmed capacity for multi-substrate use (Table 1 for details of product and substrate specificities)**. The red branch denotes TPS with C5/C10 activity, the black branches with C10/C15 activity, the blue branches with C10/C15/C20 activity, and the green branches with C15/C20 activity. These 40 multi-substrate terpene synthase are from different TPS families including TPS-a, TPS-b, TPS-g, TPS-d, TPS-e, and TPS-f. The tree was constructed by MEGA5 software by UPGMA method (Tamura et al., 2011). The asterisks denote the presence of the conserved arginine-rich RRx8W motif at the N-terminal of the protein that is common in many monoterpene synthases (Chen et al., 2011). The underlined enzymes demonstrate the presence of transit peptide.

After these first reports of multi-substrate use, knowledge of plant TPSs capable of forming terpenes of different chain length depending on substrate has been steadily increasing, and to our knowledge, there are by now at least 40 confirmed cases of multi-substrate use among plant terpenoids (Table 1). Additionally enzymes that can use C10 and C15 substrates as all the synthases mentioned above, there are enzymes that can use C5 and C10 substrates (simultaneous hemi- and monoterpene synthase activities), C10–C20 substrates (simultaneous mono-, sesqui,- and diterpene synthase activities), and C15 and C20 activity (simultaneous sesqui- and diterpene synthase activities; Table 1, Figure 2). Among these different synthases, about 80% belong to C10/C15 multi-substrate enzymes (Figure 2), and most seem to be functionally active in the cytosol as the chloroplast-targeting peptide (or mitochondrial-targeting peptide) is present only in less than one third of the proteins (12 proteins, Table 1, Figure 2). Although the putative transit peptide might be present, the homology of transit peptides is generally low, making it difficult to predict the actual subcellular targeting, and thus, its presence does not constitute the absolute proof or protein targeting to plastids (Aharoni et al., 2004). Immunolabeling and generation of fluorescent fusion proteins can ultimately solve the issue with localization, but such studies have been rare in multi-substrate enzymes (Huang et al., 2009; Carrie and Small, 2013).

TPSs with confirmed capacity of multi-substrate use are widely divergent coming from six terpene synthase families (TPS-a, TPS-b, TPS-d, TPS-g, TPS-e, TPS-f) further underscoring that the capacity for multi-substrate use could be much more widespread than previously thought. For instance, detailed examination of TPS **substrate specificities** have indicated existence of at least 9 multi-substrate TPSs in *Vitis vinifera*, out of which, two are C10/C15/C20 and the others C10/C15 synthases (Table 1). Analogously, in *Santalum* species, there are seven confirmed C10/C15 multi-substrate TPSs (Table 1), and we argue that the capacity for multi-substrate use is likely widespread across TPS families in multiple species.

KEY CONCEPT 4 **Substrate specificity.**

Enzyme affinity for different substrates. Enzymes with high substrate specificity can use only one or a few very similar substrates, while enzymes with broad substrate specificity can use a range of substrate molecules. Although the enzymes with broad substrate specificity can use multiple substrates, the enzyme affinities for different substrates typically vary as indicated by varying reaction rates with different substrates.

Previously, multi-substrate use has been associated with the lack of RRx8W motif (Aharoni et al., 2004) that is a conserved motif associated with monoterpene formation, in particular with isomerization of GDP to 3S-linalyl diphosphate and formation of cyclic products (Dudareva et al., 2003; Chen et al., 2011). However, among the multi-substrate enzymes, 11 enzymes, one C5/C10, and the others C10/C15 multi-substrate enzymes contained this motif (Figure 2). All of the RRx8W motif-containing TPSs belonged to TPS-b terpene clade with the exception of one synthase that was in TPS-a clade (Figure 2). Lack of RRx8W motif in a large number of TPSs with C10 activity, many of which do form cyclic products, suggests important modifications in catalytic mechanisms in these multi-substrate enzymes.

Although multi-substrate use is likely widespread, not all TPSs are multi-substrate synthases. In fact, steric limitations and configuration of the active center and overall protein flexibility as driven by the tertiary protein structure might rule out the use of multiple substrates. For instance, incubation of gray poplar (*Populus* x *canescens*) isoprene synthase with the larger substrate GDP demonstrated that the active center was too small to use GDP as a substrate for monoterpene production, although GDP was a competitive inhibitor of isoprene synthesis (Köksal et al., 2010). The only known enzyme capable of using both C5 and C10 substrates is a myrcene synthase in *Humulus lupulus* that can produce both monoterpenes and isoprene (Sharkey et al., 2013). In *P. x canescens* isoprene synthase, there are two phenylalanine (Phe) residues, F338 and F485 that play an important role in the functioning of the protein as obligate isoprene synthase (Sharkey et al., 2005; Köksal et al., 2010). This second Phe residue in *P. x canescens* isoprene synthase seals the H-helix side of the active site, thereby making the active site effectively smaller and avoiding the catalytic activity with GDP (Gray et al., 2011). The myrcene synthase from *H. lupulus* only possess a Phe residue homologous to F338 in *P. x canescens* and does not possess the second Phe residue homologous to F485 and instead of it, there is a Val residue (V502), allowing for accommodation of both DMADP and GDP (Sharkey et al., 2013).

Analysis of active site volumes of different TPSs based on available crystal structures does demonstrate that the active center cavity size and substrate and product sizes are closely related (Köksal et al., 2011b). Nevertheless, the active site is generally somewhat larger than the corresponding substrate molecules; in several cases, the active site cavity volume is much greater than substrate and product molecules (Köksal et al., 2011b). Obviously, enzymes with cavities tailored to their correspondent substrates can unlikely use larger substrates, while enzymes with a too large cavity might not initiate the first steps of catalysis with smaller substrates. As with the myrcene synthase in *H. lupulus* (Sharkey et al., 2013), the capacity to use multiple substrates might be associated with replacement of one or a few amino acid residues in conserved region(s), allowing for the active site of the enzyme to accept more than one substrate in alternative conformations.

## Specificity of use of different substrates among multi-substrate enzymes

Among the multi-substrate enzymes, there is a significant variation in enzyme affinity toward different substrates and overall specific activity. The C5/C10 myrcene synthase of *H. lupulus* has a lower isoprene synthase activity than other, only isoprene synthesizing enzymes (Sharkey et al., 2013), suggesting a less optimal active site structure for isoprene synthesis. In the case of C10/C15 multi-substrate *S. lycopersicum* germacrene C synthase, the sesquiterpene synthase activity exceeded the monoterpene synthase activity by a factor of ten when measured in the same enzyme preparation at saturating levels of the prenyl diphosphate substrates and Mg^2+^ cations (Colby et al., 1998). On the other hand, cucumber (*Cucumis sativus*) (*E*,*E*)-α-farnesene synthase catalyzed the formation of (*E*)-β-ocimene from GDP with similar efficiency as formation of (*E*,*E*)-α-farnesene from FDP (Mercke et al., 2004). In addition, both the cytosolic (FaNES1) and chloroplastic or mitochondrial (FaNES2) nerolidol synthases in strawberry (*Fragaria ananassa*) produced almost similar amounts of the monoterpene linalool and the sesquiterpene nerolidol from their corresponding substrates (Aharoni et al., 2004). Furthermore, two sesquiterpene synthases, (*E*)-α-bergamotene synthase from lavender (*Lavandula angustifolia*; Landmann et al., 2007), and yarrow (*Achillea millefolium*) (AmGAS; Pazouki et al., 2015) had greater affinities to GDP than to FDP when both substrates were provided in equimolar concentrations. In the case of two C10/C15/C20 enzymes, *Phaseolus lunatus* TPS (PlTPS2) and MtTPS3 from *Medicago truncatula* (Table 1), substrate specificities also widely differed. For equimolar substrate mixtures of GDP, FDP, and GGDP, the rate of product formation for PlTPS2 was the largest for the C10 compound linalool, followed by the C15 compound (*E*)-nerolidol (82% of the rate of linalool synthesis), and the C20 compound (*E*,*E*)-geranyllinalool (16% of the rate of linalool synthesis; (Brillada et al., 2013)). In contrast, for MtTPS3, the rate of formation was the greatest for (*E*)-nerolidol, followed by (*E*,*E*)-geranyllinalool (65% of the rate of (*E*)-nerolidol synthesis) and linalool (5% of the rate of (*E*)-nerolidol synthesis; Arimura et al., 2008). Such differences in the affinity for different substrates further indicate that the active center size and structure and protein tertiary structure likely importantly drive the capacity for multi-substrate use of different proteins.

Within a given substrate size class, several enzymes can accept also different substrates. Besides to FDP, the AmGAS can use both GDP and NDP (neryl diphosphate), whereas with GDP resulted in formation of acyclic monoterpenes, and with NDP in formation of cyclic monoterpenes (Pazouki et al., 2015). Thus, substrate structure importantly controlled the product profiles of AmGAS (Pazouki et al., 2015). Analogously, santalene synthase (SaSSy) from *Santalum album* was able to accept both (*E*,*E*)-FDP (*trans* isomer), (*Z*,*Z*)-FDP (*cis* isomer), and GDP as substrates (Jones et al., 2011). However, differently from AmGAS, sesquiterpene mixture produced with (*E*,*E*)-FDP and (*Z*,*Z*)-FDP differed only moderately (Jones et al., 2011; Table 1). There are monoterpene synthases that can use NDP instead of GDP, e.g., a tomato (*S. lycopersicum*) monoterpene β-phellandrene synthase expressed in glandular trichomes (Schilmiller et al., 2009). Sesquiterpene synthases that can use *Z*,*Z*-FDP instead of the usual *E*,*E*-FDP, e.g., a santalene and bergamotene synthase in wild tomato (*S. habrochaites*) have also reported (Sallaud et al., 2009). However, these *cis*-substrate using enzymes cannot use the *trans*-substrates. Thus, the capacity to use both the *cis*-and *trans*-substrate isomers in the two multi-substrate enzymes, AmGAS and SaSSy suggests a very high plasticity of the active centers of these enzymes. Such a high active center plasticity might be a more general feature of multi-substrate enzymes, but non-canonical substrates, NDP and (*Z*,*Z*)-FDP are less frequently used in functional assays than GDP and (*E,E*)-FDP. Clearly more work is needed to gain insight into the possible use of *cis*-substrates across TPSs.

## Evolution of multi-substrate use and subcellular compartmentalization of multi-substrate enzymes

**Figure 3:**
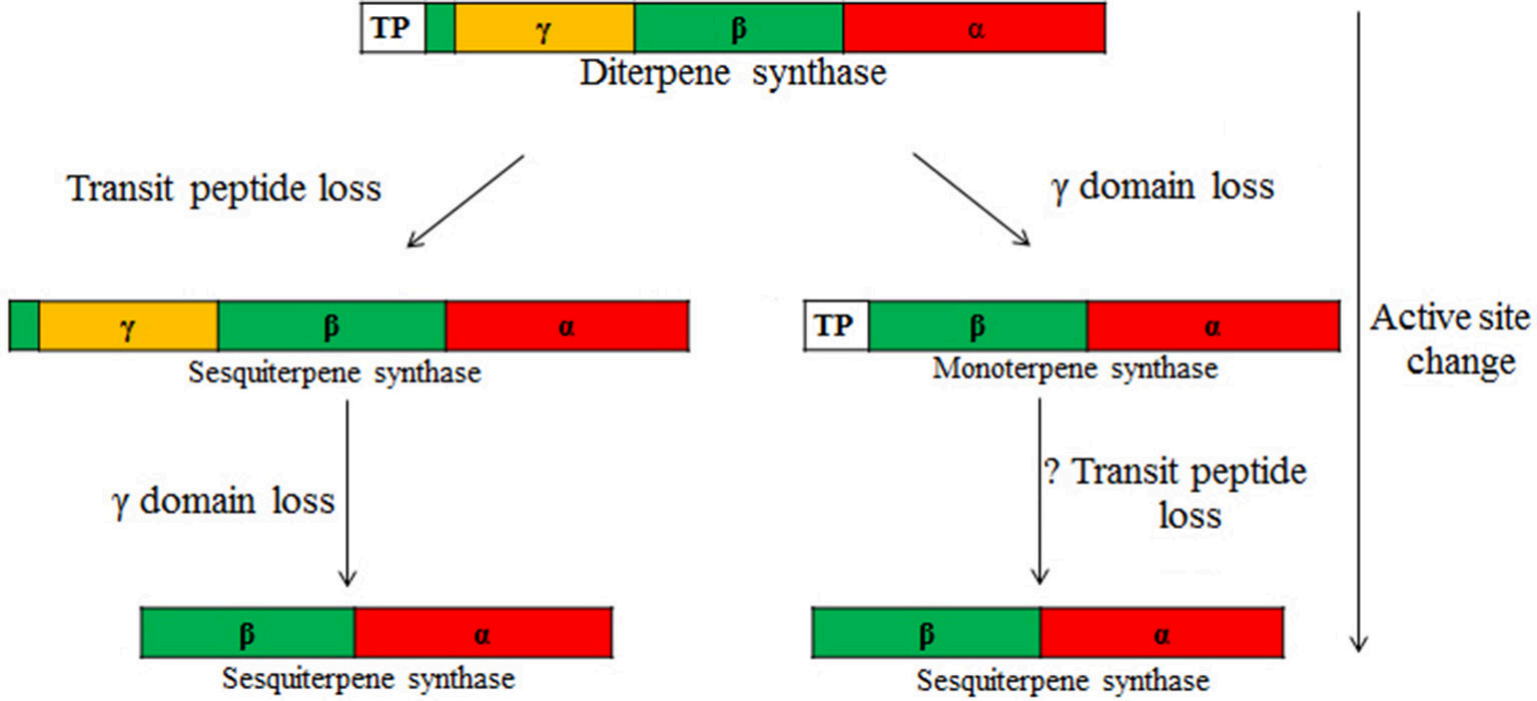
**Hypothesis of the evolution of multi-substrate enzymes according to two potential routes**. Ancient terpenoid synthases underlying the diversity of terpene synthases in plants are tri-domain, α-, β,- and γ-domain proteins with two active sites, one in the α-domain (class I activity) and the other in the β-domain (class II activity) (Christianson, 2006, 2008; Köksal et al., 2011a,b). The γ-domain without an active site is inserted between the first and second helices of the β-domain (Köksal et al., 2011a,b). These ancient proteins also carry a transit peptide (TP) at the N terminus targeting these proteins to chloroplasts. Through evolution, these complex enzymes have undergone considerable simplification, resulting in changes in catalysis, enzyme subcellular localization, and product and substrate specificities. Class II activity seems to have been lost first (not shown in the figure) and is missing in all confirmed multi-substrate enzymes. A tri-domain terpene synthase functionally active in the cytosol is formed through the loss of the transit peptide from a diterpene synthase. This can be eventually followed by γ-domain loss, resulting in formation of a bi-domain cytosol-active synthase (left). While the transit peptide is maintained, γ-domain loss can first lead to formation of a bi-domain diterpene synthase (e.g., *ent*-kaurene synthase like synthase in *Triticum aestivum*, Figure 2, Table 1) and ultimately to a monoterpene synthase. Loss of the transit peptide can further lead to a cytosol-active enzyme (e.g., β-ocimene synthase, AtTPS02, and (*E,E*)-α-farnesene synthase, AtTPS03, from *Arabidopsis thaliana* that differ in the subcellular localization due to presence or lack of the transit peptide; Figure 2, Table 1). Changes in substrate specificity are typically also associated with changes in active center size (Köksal et al., 2011b), and thus, the capacity for the use of multiple substrates will critically depend on whether the active center cavity can accommodate substrates of varying size.

Phylogenetic analyses indicate that the confirmed multi-substrate enzymes are diffusely spread across different terpene families, indicating a strong convergent nature of this trait (Figure 2), and overall demonstrating a high flexibility for evolution of enzymes with new subcellular compartmentalization and substrate specificity. It has been suggested that the demand for gibberellin production has given rise to the large superfamily of plant terpenoids (Peters, 2010), and thus, all plant terpenoid synthases are believed to originate from an ancient diterpene synthase (Hillwig et al., 2011; Köksal et al., 2011b; Rajabi et al., 2013). These phylogenetically old diterpene synthases are tri-domain, alpha-beta-gamma, proteins that contain a transit peptide (Figure 3; Hillwig et al., 2011; Köksal et al., 2011b; Rajabi et al., 2013). Further evolutionary modifications leading to diversification of product profiles have not only been associated with changes in active center structure, but isoprene and monoterpene synthases have lost the gamma-domain, while sesquiterpene synthases the **target peptide** and in most cases the gamma-domain (Hillwig et al., 2011; Köksal et al., 2011b; Rajabi et al., 2013). Existence of proteins with mixed substrate specificity allows for developing novel hypotheses about timing of major evolutionary modifications, the loss of γ-domain and transit peptide, in TPSs with different substrate specificity (Figure 3). Analysis of the structure of bi-domain, α-β, kaurene like diterpene synthase from *Triticum aestivum* (TaKSL5) that can use both *ent*-copalyl diphosphate to produce *ent*-kaurene and (*E,E*)-FDP to produce (*E*)-nerolidol (Hillwig et al., 2011), suggests that evolution of sesquiterpene synthesis can occur first by loss of γ-domain followed by changes in subcellular localization by loss of transit peptide and further diversification and loss of capacity for use of C20 substrate. Such a possibility is underscored by occurrence of multi-substrate (*E*)-nerolidol/(*E,E*)-geranyllinalool synthases in *V. vinifera* (VvPNLNGl1-VvPNLNGl4 and VvCSENerGl) that have both C15 and C20 substrate use capacity, but lack both the γ-domain and the transit peptide (Martin et al., 2010).

KEY CONCEPT 5 **Target peptide.**

Only a minority of proteins functionally active in the chloroplast are encoded by the chloroplast genome, while the majority of them is encoded by the nuclear genome. These latter proteins are translated in the cytosol, and transported into the chloroplast. These proteins typically have an N-terminal peptide that targets these proteins to chloroplast. Analogously, proteins might carry a targeting signal peptide that targets them to mitochondria. Various computer models have been constructed to predict actual subcellular targeting of given protein based on target peptide sequence, e.g. Predotar (https://urgi.versailles.inra.fr/predotar/predotar.html).

In TPS-e (TaKSL5) and TPS-f (VvPNLNGl1 and VvCSENerGl, Figure 2) clades, there are three putative intermediates of evolution of sesquiterpene synthases directly from diterpene synthases by γ-domain loss predicted to occur first followed by loss of transit peptide. On the other hand, in the TPS-d family, all the three *A. grandis* C10/C15 multi-substrate TPSs lack the transit peptide, but (*E*)-α-bisabolene synthase is a tri-domain, α-β-γ, protein, while δ-selinene and γ-humulene synthases are bi-domain, α-β, proteins (Bohlmann et al., 1998a). This suggests that in evolution toward sesquiterpene synthesis in TPS-d family proteins, the transit peptide could have been lost first, followed by the loss of the γ-domain (Figure 3).

In TPS-a, TPS-b and TPS-g families, there is evidence of evolution of sesquiterpene synthases from monoterpene synthases (Figures 2, 3). It has been suggested that *L. angustifolia* (*E*)-α-bergamotene synthase (LaBERS) has evolved from a monoterpene synthase by the loss of the plastidial signal peptide and by broadening its substrate spectrum (Landmann et al., 2007). LaBERS is similar to an α-zingiberene synthase of sweet basil (*Ocimum basilicum*) in that the latter has greater similarity to monoterpene synthases than to sesquiterpene synthases in the TPS-a group (Landmann et al., 2007). Analogously, a vestigial activity of santalene synthases with GDP suggests that these enzymes may have evolved from a monoterpene synthase ancestor through loss of the plastid signal peptide and then adaptation of the active site to (*E*,*E*)-FDP (Jones et al., 2011). Furthermore, snapdragon (*Antirrhinum majus*) has two C10/C15 (*E*)-nerolidol/linalool synthases (AmNES/LIS-1 and AmNES/LIS-2) (Nagegowda et al., 2008), *Arabidopsis thaliana* has two C10/C15 (*E,E*)-α-farnesene/(*E*)-β-ocimene synthases (AtTPS02 and AtTPS03 (Huang et al., 2010) and *F. ananassa* has two C10/C15 (*E*)-nerolidol synthases (FaNES1 and FaNES2; Aharoni et al., 2004). In all these three cases, one synthase protein lacks the target peptide in the N terminus (AmNES/LIS-1, AtTPS03, FaNES1), while the other has it (AmNES/LIS-2, AtTPS02, FaNES2) further suggesting that sesquiterpene synthases might have evolved from monoterpene synthases (Figure 3).

This evidence collectively suggests that the loss of transit peptide might have occurred both in diterpene and monoterpene synthases. In the case of two-domain sesquiterpene synthases where the signal peptide loss occurred already in diterpene synthases either before (TPS-d multi-substrate synthases) or after the loss of γ-domain (TPS-e and TPS-f multi-substrate synthases, Figure 3), one would expect a greater degree of specialization toward C15 and less affinity toward other substrates. In contrast, sesquiterpene synthases possibly evolved from monoterpene synthases where γ-domain was lost first and then followed by loss of the signal peptide, are evolutionarily more recent and could exhibit greater substrate diversity. Testing these hypotheses will require a systematic survey of functional activity of different multi-substrate synthases with varying evolutionary history using a variety of C10-C20 substrates.

## What is the physiological significance of multi-substrate use for cytosolic and plastidic enzymes?

For C5/C10 substrate TPSs like myrcene synthase from *H. lupulus* that is localized in plastids where both DMADP and GDP are available, the situation is obviously simple as such enzymes can produce both C5 and C10 products under physiological conditions with the product share depending on the relative enzyme affinity to different substrates and on the ratio of substrate availabilities. Analogously, for plastidic enzymes with C10/C20 activity as the *P. lunatus* TPS, PlTPS2, and its homolog MtTPS3 in *M. truncatula* (Table 1), presence of both substrates GDP and GGDP in plastids implies that these enzymes can form both C10 and C20 products *in vivo* without any need for substrate exchange between chloroplasts and cytosol.

The situation is less clear for C10/C15 and C15/C20 multi-substrate enzymes due to differences in subcellular localization of TPSs and corresponding substrates. Although the evidence summarized indicates that synthases capable of making monoterpenes in cytosol and sesquiterpenes in plastids are available, the key question is whether the substrates for their synthesis are available or could become available under certain conditions. If the answer is positive, the major physiological implication is that the product specificity of terpenes *in vivo* is determined by the TPS activities and relative sizes of their respective substrate pools. So far, the possibility of production of monoterpenes in cytosol and sesquiterpene synthesis in plastids by multi-substrate enzymes has been considered physiologically irrelevant due to low cytosolic pools of GDP and low plastidic pools of FDP (Tholl et al., 2004). However, there are several lines of evidence suggesting that the current consensus might need revision (Gutensohn et al., 2013; Rasulov et al., 2015; Dong et al., 2016).

In particular, there is now evidence of a certain GDP availability and consumption in each relevant compartment, cytosol, plastids and mitochondria (Dong et al., 2016). Although GDP is presumably synthesized only in plastids, it can be transported from plastids to cytosol and to mitochondria (Bick and Lange, 2003; Dong et al., 2016). A ^13^C-labeling study has demonstrated that chloroplast-derived GDP can be used in cytosolic sesquiterpene synthesis in chamomile (*Matricaria recutita*; Adam and Zapp, 1998; Adam et al., 1999), but the question is whether it can be also used for synthesis of monoterpenes in cytosol? In fact, overexpression of the multi-substrate α–zingiberene synthase (ZIS) in tomato fruits led to unpredicted formation of monoterpenes (Davidovich-Rikanati et al., 2008). Since the ZIS gene sequence is without a transit peptide, and thus, is present in the cytosol, the production of monoterpenes in the transgenic tomatoes suggests that a cytosolic GDP pool for monoterpene formation must be available (Davidovich-Rikanati et al., 2008). However, monoterpene synthesis in cytosol was relatively low unless chloroplastic GDP pool was strongly enhanced by overexpressing plastidic GDP synthase (Gutensohn et al., 2013). This evidence suggests that accumulation of chloroplastic GDP can enhance GDP transport from chloroplast to cytosol, thereby increasing the synthesis of cytosolic monoterpenes. Such an enhanced availability can be particularly relevant given the competitive inhibition of monoterpene synthase activity by cytosolic FDP that shifts the TPS reaction toward sesquiterpene synthesis in conditions of low cytosolic GDP availability.

Both biotic and abiotic stresses can potentially significantly perturb the isoprenoid metabolism, especially when MVA and MEP pathways are differently affected by given stress, potentially altering the cross-talk between isoprenoid synthesis pathways (Rasulov et al., 2015). There is some evidence demonstrating certain cooperativity between two terpene synthesis pathways under conditions leading to decreases in the activity of one of them (Piel et al., 1998; Jux et al., 2001; Page et al., 2004; Rodríguez-Concepción, 2006), but the capacity for such a substitution of function and regulation is insufficiently understood. In fact, several multi-substrate enzymes are stress-inducible, including herbivore-inducible rice (*Oryza sativa*) C10/C15 cytosolic enzyme Os08g07100 that forms zingiberene and β-sesquiphellandrene with FDP and β-myrcene with GDP as the main products (Yuan et al., 2008). Enhancement of terpene synthesis in secretory cells of aromatic plants is a common response to a variety of abiotic stresses such as drought that curbs the rate of plant growth and reduces the sink activity (Gershenzon, 1984). Capacity to form monoterpenes in the cytosol could be especially advantageous when the plastidial supply of substrate is limited due to reduced rate of photosynthesis in stressed plants. In fact, in many aromatic plants such as *A. millefolium*, mono- and sesquiterpene contents of the essential oil are strongly correlated (Mockute and Judzentiene, 2003; Orav et al., 2006; Gudaityte and Venskutonis, 2007; Judzentiene and Mockute, 2010). Such a correlation might partly rely on the mixed substrate specificity of cytosolic enzymes, and consequently reflect a more important role of cytosolic monoterpene synthesis in aromatic plants (Pazouki et al., 2015).

While several pieces of evidence, in particular, cytosolic availability of GDP, suggest that cytosolic C10/C15 enzymes could produce monoterpenes under physiological conditions, the situation is less clear with potential sesquiterpene production by plastid- and mitochondria-localized C10/C15 TPSs. Such synthases are less frequently observed than corresponding C10/C15 cytosolic enzymes (Table 1), and furthermore, the information of possible FDP availability in different subcellular compartments is limited. Initially, FDP synthesis was presumed to occur only in cytosol, but there is increasing evidence of widespread occurrence of FDP synthases targeted to mitochondria in different organisms including plants (Cunillera et al., 1997; Martín et al., 2007). An immunocytochemical study has also localized FDP synthases in chloroplasts of *O. sativa, T. aestivum*, and *Nicotiana tabacum* (Sanmiya et al., 1999). However, a plastidic FDP is apparently not present in several other species (Cunillera et al., 1997; Hemmerlin et al., 2003). Chloroplasts can exchange IDP, GDP, and FDP (Bick and Lange, 2003; Rolland et al., 2012), but the IDP/GDP/FDP transporter discovered has been suggested to support only unidirectional, chloroplasts to cytosol transport (Bick and Lange, 2003). Yet, other evidence suggests that at least the exchange of IDP is completely bidirectional (De-Eknamkul and Potduang, 2003; Laule et al., 2003; Bartram et al., 2006; Rodríguez-Concepción, 2006; Rasulov et al., 2015). Furthermore, uptake of exogenous FDP and use for chloroplastic diterpene synthesis and prenylation of chloroplast proteins has been demonstrated (Nabeta et al., 1995; Parmryd et al., 1999; Karunagoda and Nabeta, 2004). This evidence and the evidence of GDP transport summarized above suggest that there might be additional bidirectional or unidirectional cytosol-to-chloroplast transporters for GDP and FDP. Transport of corresponding alcohols, geraniol, and farnesol and presence of a chloroplastic alcohol phosphorylating system has also been considered plausible (Parmryd et al., 1999).

On the other hand, existence of bi-functional FDP/GGDP synthases is widespread across multiple organisms (Szkopinska and Plochocka, 2005; Ling et al., 2007; Jordão et al., 2013). In the case of plants, a bi-functional FDP/GGDP synthase was discovered in *Zea mays* (Cervantes-Cervantes et al., 2006) that, however, seems to be targeted to cytosol. On the other hand, mutational studies indicate that the product specificity, GGDP vs. FDP, of plastidic GGDP synthases can be achieved by only minor changes in protein sequence (Kojima et al., 2000). Indeed, big sagebush (*Artemisia tridentata*) has a plastidial bifunctional multi-substrate prenyltransferase, FDS-5, that is homologous to other FDP synthases (Hemmerlin et al., 2003). However, it does not form FDP, but catalyzes the formation of C5 and C10 substrates (Hemmerlin et al., 2003). At any rate, information about FDP synthases is surprisingly limited for many important plants, calling for further systematic studies on prenyltransferase subcellular distribution and substrate specificity.

Although the information of FDP availability in chloroplasts is limited, several pieces of evidence suggest that sesquiterpene synthesis can potentially occur in plastids. Dudareva et al. (2005) demonstrated that in fosmidomycin-treated snapdragon (*A. majus*) petals, not only monoterpene emission was inhibited, but also nerolidol emission, although the inhibitory effect was not as rapid as that for monoterpenes. This is relevant as fosmidomycin is a specific inhibitor of the plastidial MEP pathway enzyme, 1-deoxy-D-xylulose-5-phosphate reductoisomerase (DXR; Kuzuyama et al., 1998). On the other hand, snapdragon flowers treated by mevinolin, a specific inhibitor of 3-hydroxy-3-methyl glutaryl-CoA reductase (HMGR), the key enzyme of the mevalonate pathway, had almost no influence on the quantity of emitted nerolidol, suggesting that the mevalonate pathway does not contribute to nerolidol production in snapdragon flowers (Dudareva et al., 2005). Given the presence of two C10/C15 enzymes, the cytosolic AmNES/LIS1 and plastidic AmNES/LIS2 (Table 1), these results could be interpreted as indicative of transport of plastidic isoprenoid precursors to the cytosol and sesquiterpene synthesis by the cytosolic enzyme. However, with purified snapdragon leucoplasts, presence of both linalool/nerolidol synthase activities of AmNES/LIS2 (Table 1) in plastids was shown (Nagegowda et al., 2008), indicating that sesquiterpene synthesis does not necessarily require transport of plastidic metabolites to cytosol.

In addition, activation of multi-substrate plastidic C10/C15/C20 TPSs PlTPS2 in *P. lunatus* and MtTPS3 in *M. truncatula* in herbivore-infected leaves was associated with the release of both 4,8-dimethyl nona-1,3,7-triene (DMNT), which is produced from the C15 precursor (*E*)-nerolidol, and 4,8,12-trimethyl trideca-1,3,7,11-tetraene (TMTT), which is produced from the C20 precursor (*E*,*E*)-geranyllinalool (Arimura et al., 2008; Tholl et al., 2011; Brillada et al., 2013). This again suggests that the C15 precursor (*E*)-nerolidol was formed in the plastids. On the other hand, wounding and methyl jasmonate induction of the tomato (*S. lycopersicum*) plastidic TPS LeMTS1 that has both linalool synthase (GDP) and (*E*)-nerolidol synthase (FDP) activities was only associated with increased linalool emissions but did not change nerolidol levels (Van Schie et al., 2007), again suggesting that substrate availability for sesquiterpene synthesis might ultimately limit sesquiterpene synthesis by plastidic C10/C15 TPSs.

## Conclusions

Our analysis suggests that multi-substrate use is more common in plants than generally thought and advocates for conduction of further systematic studies using multiple substrates across phylogenetically different plant groups harboring TPSs from different clades to gain an insight into the existence of the capacity for multi-substrate use across plant kingdom. While C5/C10 and C10/C20 multi-substrate plastidic enzymes can readily catalyze formation of multiple products because their C5, C10, and C20 substrates are available in the plastids, this review also challenges the widespread consensus that presence of GDP and monoterpene synthesis is confined to the plastids, and presence of FDP and sesquiterpene synthesis is confined to the cytosol. In particular, recent evidence suggests that plastidic GDP can support monoterpene synthesis in cytosol (Davidovich-Rikanati et al., 2008; Gutensohn et al., 2013; Pazouki et al., 2015; Dong et al., 2016) and potentially even in mitochondria (Dong et al., 2016). The situation with the presence of FDP and sesquiterpene synthesis in plastids is less clear, reflecting the limited information of subcellular localization of FDP synthases in plants and existence of a capacity for formation of products of different chain length in plant prenyltransferases. Nevertheless, several pieces of evidence suggest that FDP could at least be transported from cytosol into plastids, potentially supporting sesquiterpene synthesis by multi-substrate enzymes there. The overall significance of alternative activities of multi-substrate enzymes will critically depend on the enzyme specificity and relative availability for different substrates. Perturbation of terpenoid metabolism under stress conditions can lead to enhanced substrate exchange between cytosol and plastids (Rasulov et al., 2015) as well as modifications in the expression of enzymes responsible for product pool sizes (Steele et al., 1998), and thus, favor synthesis of terpenoids according to non-conventional pathways.

## Author contributions

All authors listed, have made substantial, direct and intellectual contribution to the work, and approved it for publication.

### Conflict of interest statement

The authors declare that the research was conducted in the absence of any commercial or financial relationships that could be construed as a potential conflict of interest.
